# Beyond the Golden Hour: Association of Prehospital Time and Intervention Quality With Functional Outcomes in Severely Injured Trauma Patients

**DOI:** 10.7759/cureus.112545

**Published:** 2026-07-12

**Authors:** Shourya Vijayvargia, Nishith S Mandal, Indu B Dubey, Akshita Akshita, Aashutosh M Hiremath, Aakansha Giri Goswami, Dilpreet Singh Dhillon, B Harish Reddy, Dhanesh Sipani, Ayush Baldania

**Affiliations:** 1 Department of Surgery, Vardhman Mahavir Medical College and Safdarjung Hospital, New Delhi, IND

**Keywords:** advanced trauma life support (atls), emergency medical services, low- and middle-income countries (lmic), prehospital care, trauma

## Abstract

Background: Trauma remains a major cause of mortality and disability worldwide, particularly in low- and middle-income countries (LMICs). Although the “golden hour” concept emphasises rapid transport after injury, emerging evidence suggests that the quality of prehospital care may influence outcomes more than transport time alone. Data evaluating the relationship between prehospital care and trauma outcomes in low-resource settings remains limited.

Objective: To evaluate the association between prehospital time and prehospital treatment with outcomes in severely injured trauma patients presenting to a tertiary care trauma centre.

Methods: This prospective observational cohort study was conducted in the Department of General Surgery at a tertiary care centre over 18 months. Seventy-five severely injured trauma patients aged more than 12 years with Revised Trauma Score (RTS) less than 4 or other criteria for severe trauma were included. Data regarding demographics, mechanism of injury, mode of transport, prehospital time, and prehospital interventions were collected using a predefined proforma. Prehospital interventions were categorised as none, non-Advanced Trauma Life Support (ATLS)-compliant, or ATLS-compliant. Primary outcomes included 30-day mortality, hospital length of stay, and time to return to work. Statistical analysis was performed using Chi-square or Fisher's Exact tests as appropriate, with p<0.05 considered significant.

Results: The mean age of participants was 33 years, and 62 (83%) were male. Road traffic injuries were the most common mechanism of trauma (38, 51%). Most patients reached the tertiary centre within one to three hours of injury (25, 33%). ATLS-compliant prehospital intervention was received by only 20 (27%) patients.

Overall mortality was observed in seven (9%). Prehospital time was not significantly associated with mortality, hospital stay duration, or return to work. However, ATLS-compliant prehospital intervention was significantly associated with shorter hospital stay and earlier return to work. No statistically significant association was observed between prehospital intervention and mortality.

Conclusion: Prehospital intervention quality, rather than prehospital transport time alone, was significantly associated with better functional recovery in severely injured trauma patients. ATLS-based prehospital stabilisation was associated with shorter hospitalisation and earlier return to work. These findings support strengthening structured prehospital trauma systems and protocol-driven emergency care in low-resource settings.

## Introduction

Trauma remains a major global public health challenge and is among the leading causes of mortality and disability worldwide, particularly in low- and middle-income countries (LMICs). Road traffic injuries (RTIs), falls, and interpersonal violence collectively account for more than six million deaths annually, with nearly 90% occurring in LMICs [1]. Trauma, in these countries, contributes substantially to mortality among the economically productive population, resulting in major socioeconomic consequences due to prolonged disability, loss of income, and increased healthcare expenditure [2].

The prehospital phase of trauma care plays a critical role in determining patient outcomes. Early recognition, resuscitation, haemorrhage control, airway management, and rapid transfer to definitive care are central principles of modern trauma systems. The concept of the “golden hour,” introduced by Cowley in the 1970s, emphasised that definitive intervention within the first hour following injury could significantly improve survival [3]. However, contemporary evidence suggests that the quality and appropriateness of prehospital interventions may be more important than transport time alone [4].

Several recent studies have questioned the universal applicability of the golden hour concept. A large multicenter Asian cohort study involving more than 24,000 trauma patients demonstrated no significant association between total prehospital time and 30-day mortality. However, prolonged prehospital intervals were associated with poorer functional outcomes at discharge [4]. Similarly, systematic reviews have reported that rapid transport may confer the greatest benefit in patients with penetrating trauma, hemorrhagic shock, and traumatic brain injury. In contrast, among patients with blunt trauma, outcomes appear to depend not only on transport time but also on the quality of prehospital stabilisation and resuscitative care delivered before hospital arrival [5-7].

Despite advances in trauma systems in high-income countries, prehospital care in many LMICs remains fragmented and underdeveloped [8]. Deficiencies in ambulance infrastructure, delayed transport, lack of trained emergency personnel, inadequate triage systems, and poor interfacility coordination continue to contribute to preventable trauma-related morbidity and mortality [9]. In LMICs, emergency medical services (EMS) remain heterogeneous, with many trauma victims transported by relatives, police personnel, or untrained bystanders without essential stabilisation measures. The absence of organised trauma registries and the limited availability of prospective data further restrict evidence-based policy development [9-11].

Emerging evidence from LMICs suggests that structured prehospital systems and protocol-driven interventions can significantly improve trauma outcomes [10,12]. A systematic review by Henry et al. demonstrated that implementation of organised prehospital trauma systems reduced mortality by approximately 25% in developing countries [10]. Similarly, studies evaluating trauma notification systems and protocol-driven EMS pathways have demonstrated improvements in hospital preparedness, trauma team activation, treatment efficiency, and overall trauma system performance [12,13].

Most available trauma literature, however, focuses primarily on mortality as the principal endpoint. Limited data exist regarding functional recovery outcomes such as duration of hospital stay, post-trauma complications, and return to work, particularly in LMIC settings. These outcomes are especially relevant in younger trauma populations, where delayed functional recovery can have profound economic and social consequences.

Furthermore, there remains a paucity of prospective data evaluating the relationship between prehospital time, quality of prehospital intervention, and trauma outcomes in severely injured patients in LMICs. Understanding this relationship is essential for strengthening trauma systems, optimising referral pathways, and developing context-specific EMS protocols in resource-constrained settings.

Therefore, the present study aimed to evaluate the association between prehospital transport time and the quality of prehospital intervention with functional and clinical outcomes among severely injured trauma patients presenting to a tertiary trauma centre in an LMIC. We hypothesised that the quality of prehospital intervention would demonstrate a stronger association with patient outcomes than transport time alone. The primary outcomes were 30-day mortality, hospital length of stay, and time to return to work. Secondary outcomes included post-trauma complications, intensive care unit admission, requirement for ventilatory support, and operative intervention.

## Materials and methods

Study design and setting

This prospective observational cohort study was conducted in the Department of General Surgery at Vardhman Mahavir Medical College and Safdarjung Hospital over 18 months, following approval from the Institutional Ethics Committee (IEC/VMMC/SJH/Cert/02-2024/144). The study evaluated the association of prehospital time and prehospital interventions with outcomes in severely injured trauma patients presenting to a tertiary care trauma centre.

Study population

All trauma patients aged 12 years or older who presented to the emergency department and were admitted to the Department of General Surgery were screened for eligibility.

Inclusion criteria

Patients were eligible for inclusion if they had severe trauma, defined by a Revised Trauma Score (RTS) of less than 4 or the presence of a serious injury requiring admission. Serious injury was defined as involvement of two or more body systems or body regions, abnormal vital parameters suggestive of physiological compromise, requirement for surgical intervention within 24 hours of presentation, high-velocity or other dangerous mechanisms of injury, hemorrhagic shock requiring blood transfusion and operative control, or life-threatening conditions such as airway obstruction, tension pneumothorax, cardiac tamponade, massive hemothorax, or unstable pelvic fractures.

Exclusion criteria

Patients who left against medical advice, were lost to follow-up, or were transferred to other speciality departments were excluded from the study.

Sample size

The sample size was calculated using the formula for estimation of a single population proportion:

\[ N = \frac{Z_{1-\alpha/2}^{2}\, p(1-p)}{d^{2}} \]

where *N* is the required sample size, $Z_{1-\alpha/2}$ is the standard normal deviate corresponding to a 95% confidence level (1.96), *p *is the estimated prevalence of in-hospital mortality among major trauma patients (24%) based on the study by Dharap et al. [14], and* d* is the absolute precision (10%).

Using these assumptions, the minimum calculated sample size was 70 patients. After accounting for a 5% attrition rate, the final required sample size was 74 patients.

Study procedure

All eligible patients underwent initial assessment and resuscitation in accordance with Advanced Trauma Life Support (ATLS) principles [15]. Primary survey and resuscitation were performed with emphasis on airway stabilisation, breathing assessment, circulatory support, neurological evaluation, and exposure with environmental control.

Following stabilisation, a secondary survey was performed, including a detailed systemic examination and collection of trauma-related history using the AMPLE framework (Allergies, Medications, history, Last meal, and Events related to injury).

Clinical and demographic data were recorded using a predefined proforma. Information collected included the mechanism and intent of injury, mode of transportation, prehospital interventions received, transport and referral timelines, treatment provided at referring facilities, and interventions performed after arrival at the tertiary trauma centre. Prehospital information was obtained from ambulance documentation, referral records, emergency department documentation, and, when necessary, interviews with the patient or an accompanying relative.

For patients presenting directly from the scene of injury, total prehospital time was recorded from the time of injury to arrival at the study centre. For patients referred from other healthcare facilities, the interval from injury to arrival at the tertiary care centre was used to assess prehospital time. 

Definitions

Prehospital intervention was categorised into three groups: no intervention, intervention not compliant with ATLS principles, and intervention compliant with ATLS principles. ATLS-compliant interventions included airway management, breathing support, haemorrhage control, establishment of intravenous access, fluid resuscitation, cervical spine immobilisation, and haemodynamic stabilisation before arrival at the tertiary care centre. The RTS was calculated using the standard weighted combination of Glasgow Coma Scale (GCS), systolic blood pressure (SBP), and respiratory rate (RR) variables [16]. Patients with an RTS of less than 4 were classified as severely injured.

Outcome measures

The primary outcomes assessed were 30-day mortality, duration of hospital stay, and time to return to work following discharge. Return to work was assessed during follow-up as the interval from hospital discharge until resumption of the patient's usual occupation or routine daily work.

Secondary outcomes included the occurrence of trauma-related complications, requirement for intensive care unit (ICU) admission, need for ventilatory support, and requirement for operative intervention during the index hospitalisation.

Statistical analysis

Data were entered into Microsoft Excel (Redmond, WA, USA) and analysed using Stata version 15.0 (StataCorp, College Station, TX, USA). Normality of continuous variables was assessed using the Shapiro-Wilk test. Continuous variables with normal distribution were expressed as mean ± standard deviation, while categorical variables were presented as frequency and percentage.

Associations between prehospital time, prehospital intervention, and outcome variables were analysed using Chi-square when the assumptions for its use were satisfied or Fisher's Exact tests when expected cell frequencies were small (expected count <5 in any cell). A p-value <0.05 was considered statistically significant.

## Results

Baseline characteristics

A total of 75 severely injured trauma patients were included in the study. Complete baseline and in-hospital outcome data were available for all patients.

The mean age of the study population was 33.1 ± 11.4 years (range: 15-62 years). Most patients were in the younger age group, with 35 patients (47%) aged below 30 years and 32 patients (43%) aged between 31 and 50 years. Only eight patients (11%) were older than 50 years.

The majority of patients were male (62, 82.7%), whereas females were 13 in number (17.3%).

RTIs were the most common mechanism of trauma, accounting for 38 patients (50.7%), followed by blunt assault (13, 17.3%) and stab injuries (13, 17.3%). Falls contributed to injuries in eight (10.7%) patients. Most injuries were non-intentional (50, 66.7%) (Table 1).

**Table 1:** Description of the History of Injury Values are presented as n (%).

History of injury	Values n (%)
Mechanism	
Road Traffic Injury (RTI)	38 (51%)
Blunt assault	13 (17%)
Stab	13 (17%)
Fall	8 (11%)
Roof/wall collapse	2 (3%)
Don’t know	1 (1%)
Intent	
Non-intentional	50 (67%)
Intentional	24 (32%)
Others	1 (1%)

Clinical characteristics at presentation

At presentation, 57 patients (76%) had tachycardia with a heart rate ≥100 beats/minute, and 43 patients (57%) had SBP <90 mmHg, suggestive of shock. RR >22 breaths/minute was observed in 67 patients (89%), and oxygen saturation below 96% was documented in 50 patients (67%). Overall, shock at presentation was present in 43 patients (57%).

Focused Assessment with Sonography in Trauma (FAST) was positive in 72 patients (96%). Mean GCS score at emergency department presentation was 14 ± 1.0. Splenic injury was the most common abdominal injury identified on computed tomography (20, 26%), followed by liver injury (14, 19%). Rib fractures were the most frequent thoracic injury identified on chest radiography (14, 19%).

Prehospital characteristics

**Figure 1:**
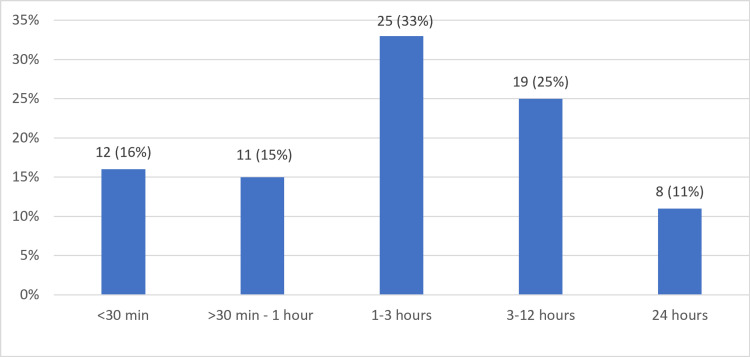
Percentage Distribution of the Study Participants as per Prehospital Transport Time

Regarding transport timelines, most patients reached the tertiary care centre within one to three hours of injury (25, 33%), followed by those reaching within three to 12 hours (19, 25%). Only 12 (16%) patients arrived within 30 minutes (Figure 1). 

Ambulances were the most common mode of transportation overall. Twenty-two patients (29%) were transported by CATS ambulance, 20 (27%) by private ambulance, and 22 (29%) arrived via self- or relative transport. Police transport accounted for 11 (15%) cases.

Prehospital interventions were absent in 44 patients (59%). ATLS-compliant prehospital interventions were documented in only 20 patients (27%), whereas 11 patients (14%) received interventions not compliant with ATLS principles.

In-hospital management and outcomes

Non-operative management was performed in 32 patients (43%), while intercostal drainage tube insertion was required in 25 patients (33%). Emergency laparotomy was performed in 14 patients (19%), and angioembolization was performed in four patients (5%).

Overall, 39 patients (52%) underwent operative intervention. ICU admission was required for 10 patients (13%), and ventilatory support for 13 patients (16%). Post-trauma complications developed in 11 patients (15%).

Seven patients (9%) died within 30 days of hospitalisation. Among survivors, 35 patients (52%) were discharged within one week, while only four patients (6%) required hospitalisation beyond four weeks. Forty-four patients (65%) returned to work within one month following discharge (Table 2).

**Table 2:** Description of Patient Outcomes

Mortality (N=75, %)	
Yes	7 (9%)
No	68 (91%)
Duration of hospital stay (N=68, %)	
<1 week	35 (52%)
1-2 weeks	18 (26%)
2-3 weeks	11 (16%)
≥ 4 weeks	4 (6%)
Time to return to work, (N=68, %)	
<1 month	44 (64.7%)
>1 month	24 (35.3%)
Post-trauma complication (N=68, %)	
Nil	57 (84%)
Yes	11 (16%)

Association between prehospital time and outcomes

No statistically significant association was observed between prehospital transport time and mortality (p=0.389), duration of hospital stay (p=0.235), or time to return to work (p=0.148).

Although patients with prolonged transport times tended to have longer hospital stays and delayed return to work, these differences were not statistically significant (Table 3).

**Table 3:** Association Between Prehospital Time and Outcomes Values are presented as n (%). Statistical significance was defined as p<0.05. No statistically significant associations were observed.

Outcome	<30 min	30 min–1 hr	1–3 hr	3–12 hr	>12 hr	Test statistic	p-value
Mortality	0	2 (28%)	1 (14%)	3 (43%)	1 (14%)	Fisher's exact	0.389
Hospital stay >2 weeks	0	1 (9%)	5 (20%)	5 (20%)	4 (50%)	Fisher's exact	0.235
Return to work >1 month	0	1 (4%)	10 (42%)	8 (33%)	5 (21%)	Fisher's exact	0.148

Association between prehospital intervention and outcomes

Prehospital intervention quality was significantly associated with functional recovery outcomes. Receipt of ATLS-compliant intervention was significantly associated with a shorter hospital stay (p<0.05) and an earlier return to work (χ² = 19.02, df 2, p<0.001) compared with patients who received no intervention or a non-ATLS-based intervention.

No statistically significant association was observed between prehospital intervention and mortality (p=0.389), although no deaths occurred among patients receiving ATLS-compliant prehospital intervention (Table 4).

**Table 4:** Association Between Prehospital Intervention and Patient Outcomes Values are presented as n/N (%). Percentages were calculated using the number of eligible patients within each intervention category as the denominator. Hospital stay and return-to-work analyses were restricted to surviving patients. ATLS = Advanced Trauma Life Support, χ² = Chi-square, df = degrees of freedom *Statistically significant at p<0.05.

Prehospital Intervention	Mortality n/N (%)	Hospital Stay >2 Weeks n/N (%)	Return to Work >1 Month n/N (%)
None (n=44)	5/44 (11.4%)	8/39 (20.5%)	10/39 (25.6%)
Non-ATLS compliant (n=11)	2/11 (18.2%)	5/9 (55.6%)	9/9 (100%)
ATLS-compliant (n=20)	0/20 (0%)	2/20 (10.0%)	5/20 (25.0%)
Test statistic	Fisher's exact	Fisher's exact	χ² = 19.02 (df 2)
p-value	0.389	<0.05*	<0.001*

## Discussion

This prospective observational study evaluated the relationship between prehospital time, prehospital intervention, and trauma outcomes among severely injured patients presenting to a tertiary trauma centre in LMICs. The findings demonstrate that while prehospital transport time alone was not significantly associated with mortality or functional outcomes, the quality of prehospital intervention, particularly ATLS-compliant stabilisation, was significantly associated with shorter hospital stay and earlier return to work. These findings contribute important prospective evidence from an LMIC setting and support the evolving concept that “what is done” during the prehospital phase may be more important than “how fast” the patient reaches definitive care.

The demographic profile of the study population reflected the global epidemiology of trauma. Most patients were young adult males, with RTIs being the predominant mechanism of injury. This observation is consistent with trauma studies, including the work of Dharap et al. and Haagsma et al., which demonstrated that trauma disproportionately affects economically productive young men [14,17]. The socioeconomic implications of this are substantial, particularly in LMICs where trauma-related disability directly impacts household income and long-term productivity.

RTIs accounted for more than half of all trauma cases in the present study, reaffirming its role as the leading contributor to severe trauma in LMICs. Similar findings have been reported by national and global injury burden studies, which attribute rising trauma mortality in LMICs to increasing urbanisation, motorisation, poor road infrastructure, and inadequate enforcement of safety regulations. The predominance of blunt trauma observed in this cohort also mirrors previously published trauma data [14].

One of the most important findings of the study was the absence of a statistically significant association between prehospital time and mortality. This finding aligns with several contemporary trauma studies that challenge the traditional interpretation of the “golden hour.” Chen et al., in the multicenter PATOS study involving more than 24,000 trauma patients across Asia, similarly reported that prolonged prehospital time was not independently associated with increased 30-day mortality [4]. However, they demonstrated worsening functional outcomes with increasing delays. Likewise, Berkeveld et al. found no significant difference in prehospital times between survivors and non-survivors among polytrauma patients [18].

These observations collectively suggest that the relationship between time and trauma outcomes is more nuanced than previously believed. Historically, the golden hour emphasised rapid transportation as the central determinant of survival. However, modern trauma research increasingly supports a physiology-based approach in which the appropriateness and quality of resuscitation may outweigh strict transport timelines [5,19]. Osterwalder et al. even demonstrated that extended prehospital intervals could be safe in blunt polytrauma when accompanied by adequate stabilisation and resuscitative measures [19]. However, these findings should be interpreted within the context of blunt trauma and do not diminish the importance of timely access to definitive care, particularly for time-critical injuries such as penetrating trauma, exsanguinating haemorrhage, or combat-related injuries.

The present study adds to the growing body of evidence suggesting that the quality of prehospital care is an important component of trauma management. ATLS-compliant prehospital intervention was significantly associated with shorter hospital stay and earlier return to work compared with patients who received no intervention or inadequate intervention. These findings suggest that greater emphasis on the quality of structured prehospital stabilisation, in addition to timely transport, may contribute to improved recovery trajectories.

These findings are supported by international literature emphasising the importance of structured prehospital care systems. Henry et al., in a systematic review and meta-analysis of trauma systems in LMICs, reported significantly reduced mortality following implementation of organised prehospital trauma systems [10]. Similarly, studies evaluating trauma notification systems and protocol-driven EMS pathways have demonstrated improvements in hospital preparedness, trauma team activation, treatment efficiency, and overall trauma system performance [13,20].

Importantly, the present study expands on existing literature by incorporating functional recovery outcomes such as return to work. Most trauma studies continue to focus almost exclusively on mortality, even though many survivors experience prolonged disability and socioeconomic impairment. In the present cohort, nearly two-thirds of survivors returned to work within one month, and earlier return to work was significantly associated with ATLS-based prehospital care. This observation is particularly relevant in LMIC settings where trauma disproportionately affects wage earners and younger populations.

The inclusion of return to work as an outcome measure strengthens the study's practical and socioeconomic relevance. Recent work by Gabbe et al. and Neubert et al. has highlighted return to work as an important marker of functional recovery after major trauma [21,22]. However, very limited prospective trauma data have incorporated such outcomes. By demonstrating that prehospital intervention quality was associated with earlier socioeconomic reintegration, the present study adds an important dimension to trauma outcome research in resource-limited settings.

Another notable finding was the poor penetration of structured prehospital trauma care. Only 27% of patients received ATLS-compliant prehospital interventions, while the majority received inadequate or no interventions. Despite ambulance transport being relatively common, there was considerable heterogeneity in the quality of care delivered before hospital arrival. This reflects broader systemic deficiencies in EMSs across LMICs, including inadequate training, fragmented referral systems, limited communication infrastructure, and the absence of standardised trauma protocols.

The findings therefore reinforce the importance of strengthening trauma systems through improvements in the quality and standardisation of prehospital care. Structured EMS training, implementation of standardised ATLS-based prehospital protocols, integration of trauma registries, development of coordinated referral pathways, and community first-responder programs may be more strongly associated with favourable outcomes than transport speed alone. Emerging LMIC models, such as the Emergency First Aid Responder (EFAR) system in South Africa and lay responder programs in Uganda, further support the feasibility of low-cost, community-based trauma interventions [23,24].

The low mortality rate observed in the study may partially explain the lack of statistical association between intervention and mortality. However, this finding should be interpreted cautiously because the study was not powered to detect differences in mortality. Additionally, neurosurgical and orthopaedic trauma patients were not included because of institutional admission pathways, potentially excluding some of the highest-risk trauma populations. Severe traumatic brain injury has been shown in previous studies to be particularly sensitive to prehospital delays and airway management quality. This may explain why some head injury-focused studies demonstrate stronger time-mortality associations than mixed-trauma cohorts.

Overall, the present study adds to the growing body of evidence suggesting that effective, protocol-driven prehospital stabilisation may be associated with improved functional recovery and should be considered alongside transport time when strengthening trauma systems, particularly in resource-constrained settings. In LMIC settings, optimising the quality of early trauma care may yield greater benefit than attempting to meet unrealistic transport timelines.

Strengths

The study has several important strengths. First, it was a prospective observational study, enabling systematic, real-time collection of trauma data. Second, unlike many previous trauma studies, the study incorporated functional recovery outcomes such as duration of hospital stay and return to work in addition to mortality. Third, the study provides valuable prospective evidence from an LMIC tertiary trauma centre, where high-quality prehospital trauma research remains limited. Finally, the study specifically evaluated the quality of prehospital interventions using ATLS principles, helping shift the focus of trauma outcome research from transport time alone to intervention quality.

Limitations

The study also has several limitations. The sample size was relatively small, limiting statistical power and reducing the generalizability of findings. Being a single-centre study conducted at a tertiary referral hospital, the findings may not represent trauma patterns and EMS systems in rural or resource-limited regions. Neurosurgical and orthopaedic trauma patients were excluded because of institutional admission practices, potentially affecting mortality and functional outcome estimates. Additionally, outcomes were primarily analysed using unadjusted statistical methods, and residual confounding from injury severity, referral bias, and comorbid conditions cannot be excluded. In addition, reliance on available prehospital documentation may have resulted in exposure misclassification, while the analysis of return to work among survivors may be subject to survivorship bias. These factors should be considered when interpreting the observed associations. Longer-term quality-of-life outcomes beyond return to work were also not evaluated. Future multicentric studies with larger cohorts and adjusted multivariable analyses are needed to validate these findings and better define the role of prehospital intervention quality in trauma recovery.

## Conclusions

This prospective observational study found that prehospital transport time alone was not significantly associated with mortality, duration of hospital stay, or return to work among severely injured trauma patients. In contrast, the quality of prehospital care, particularly ATLS-compliant stabilisation and resuscitation, may contribute to shorter hospital stay and earlier return to work. These findings challenge a purely time-centric interpretation of the “golden hour” and support the growing recognition that the effectiveness of prehospital intervention may be more important than transport speed alone.

The study highlights important deficiencies in prehospital trauma care within LMIC settings, where many patients receive inadequate or no organised prehospital intervention before reaching definitive care. Strengthening EMSs through standardised ATLS-based protocols, trained first responders, coordinated referral pathways, and robust trauma registries may improve functional recovery after severe injury. Larger multicentric studies are needed to further evaluate the impact of prehospital intervention quality on long-term clinical and socioeconomic outcomes.
